# The clinical and epidemiological evolution of varicella in Romania during 2004 and 2013

**Published:** 2015

**Authors:** A Rafila, D Pitigoi, A Arama, A Stanescu, F Buicu

**Affiliations:** * ”Carol Davila” University of Medicine and Pharmacy, Bucharest, Romania; ** ”Prof. Dr. Matei Bals” National Institute of Infectious Diseases, Bucharest, Romania,; *** ”Targu Mures” University of Medicine and Pharmacy, Targu Mures, Romania; **** National Institute of Public Health, Bucharest, Romania

**Keywords:** varicella, clinical and epidemiological evolution, communicable disease, vaccination, immunization

## Abstract

**Introduction.** Varicella, a vaccine preventable disease (VPD) is one of the most common communicable diseases in Romania.

The objectives of our study were to describe the epidemiological evolution of varicella in Romania between 2004 and 2013 and the clinical characteristics of the cases admitted to NIID between 2011 and 2013.

**Materials and methods.** An epidemiological retrospective study was conducted by using the information reported quarterly by general practitioners and hospitals at the national level.

There is no system for the surveillance of severe cases in Romania, so, to describe the clinical characteristics of varicella cases, a second retrospective study was developed, in which the patients hospitalized in the NIID, within the period 2011-2013, were included. Questionnaires were completed by using data from the clinical observation forms.

Collected information included demographic, clinical and laboratory data, complications, date of onset and admission, length of stay, admission and discharge diagnosis.

Data were processed and analyzed by using Microsoft Excel program.

**Results.** A total of 504,844 cases were reported of at the national level between 2004 and 2013, with a mean incidence of 238.2/ 100,000 inhabitants.

The most affected age group was 5-9 years old (incidence 1362.7/ 100,000 inhabitants).

The study conducted in NIID, registered 353 patients hospitalized with varicella between 2011 and 2013.

Most of the hospitalized cases (88.8%) were under 10 years old and many (72.6 %) attended a community. The majority of cases had rash (98.6%) and fever (79.9%). The main complications were pneumonia (46.2%), bacterial infection (16.1%) and encephalitis (2.5%).

**Discussions.** Varicella is a very common disease in Romania, which may develop complications. A specific surveillance system should be introduced in order to provide accurate epidemiological, clinical and laboratory information to assess whether varicella is a public health problem in Romania and if the introduction of vaccination in NIP is recommended. However, given the large number of current cases in Romania, a solution may be a sentinel surveillance system type.

## Introduction

Varicella is a vaccine preventable disease caused by varicella zoster virus. The disease is still widespread with cases occurring worldwide, especially during childhood.

Varicella is a common disease in Europe. According to EUVAC.NET [**1**], during 2000 and 2007, there were 5,435,223 varicella reported cases in 15 EU countries, that have a mandatory reporting system, with a mean incidence of 319 cases per 100,000 inhabitants. The highest incidence rates were recorded in the age group of 1-4 years old (2588 cases per 100,000 inhabitants) and 5-9 years old (1,943 cases per 100,000 inhabitants). Varicella is not included in the list of reportable diseases in the European Union, and until now, no standard case definition has been established.

According to the in force legislation in Romania, that approved the list of priority communicable diseases [**2**], varicella along with other infections (rabies, foodborne intoxications, typhoid fever, trichinosis, echinococcosis, giardiasis, leptospirosis, brucellosis, listeriosis, Q fever, botulism, toxoplasmosis, typhus, tularemia, boutonneuse fever, leishmaniasis, leprosy and yellow fever) is subject to the reporting of the disease and the application of control measures.

The vaccine against varicella is not included in the National Immunization Program in Romania, but is available on the market. Varicella vaccine has been licensed in Romania as a mono-vaccine and as combined measles-mumps-rubella-varicella (MMRV).

The objectives of our study were to describe the epidemiological evolution of varicella in Romania in the last 10 years (2004-2013) and the clinical characteristics of the cases admitted in the biggest infectious diseases hospitals in the last three years (2011-2013). Based on the clinical and epidemiological data, it could be evaluated if varicella is a national health problem and if the introducing of the vaccination in the national immunization programme is a priority.

## Materials and methods

To describe the epidemiological evolution of varicella in Romania, a retrospective study was conducted by using the information reported quarterly by the general practitioners and hospitals in the National Statistics Centre and National Centre for Surveillance and Control of Communicable Diseases [**3**-**7**], and also on the data available in literature for the period 2004-2013. Varicella cases are reported quarterly based on the clinical criteria according to age, county and area of residence. Cases of herpes zoster are not reported nationally.

The data was introduced in a database and an analysis using the Excel program was performed. The overall incidence and specific incidence by age and area of residence were calculated.

There is no system for the surveillance of severe cases and complications in Romania, so, to describe the clinical characteristics of varicella cases, a second retrospective study was developed, in which all the patients who were hospitalized in “Prof. Dr. Matei Bals” National Institute of Infectious Diseases (NIID) in Bucharest with varicella, within a period of three years (2011-2013), were included. NIID was chosen because it is the largest hospital of infectious diseases in Romania, gives specialized medical assistance for the population in Bucharest and of 6 neighboring counties.

A total of 353 patients, children and adults, from rural and urban areas, admitted with the diagnosis of varicella for at least 24 hours, were enrolled in the study. Only 10 cases had laboratory confirmation.

A questionnaire was developed to collect the data. It was completed by using the data obtained from the clinical observation form filled by physicians for each patient.

Demographic information (age, sex, county of residence, occupation, place of work), clinical signs and symptoms (fever, rash, cough, dyspnea, chest pain, vomiting, headache, neck stiffness, Kernig sign/ Brudzinsky, drowsiness, convulsions, impaired balance/ gait, hematuria, edema, jaundice), laboratory data, presence of complications (encephalitis, pneumonia, bacterial infections of the skin, cerebellar ataxia, aseptic meningitis, thrombocytopenia, purpura fulminans, myocarditis, iritis, uveitis, Reye Syndrome, Guillain-Barre syndrome, hemorrhagic varicella, arthritis, hepatitis), date of onset, date of admission, length of stay, admission diagnosis, discharge diagnosis, was collected.

Data were processed and analyzed by using Microsoft Excel program.

The study protocol was approved by the local ethics committee.

## Results

**Fig. 1:**
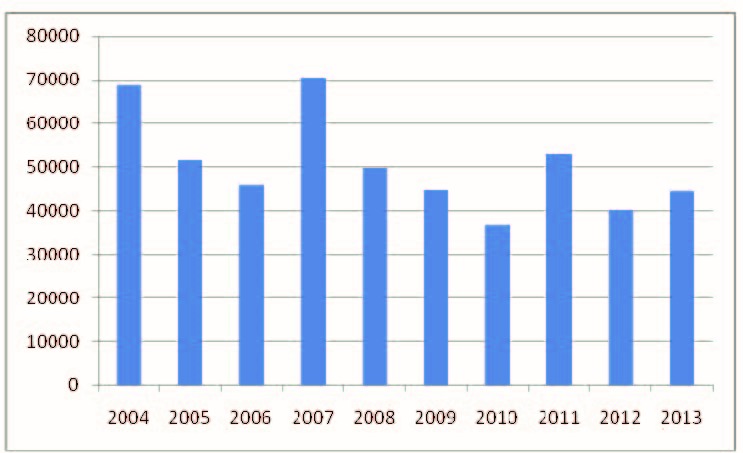
Number of varicella cases in Romania, 2004-2013

A significant number of cases of varicella are reported annually in Romania (**Fig. 1**).

There were a total of 504,844 reported cases of varicella at national level in the studied period (2004-2013). Most cases (70 410 cases) were recorded in 2007 and the lowest number of cases were registered in 2010 (36 504 cases).

**Fig. 2:**
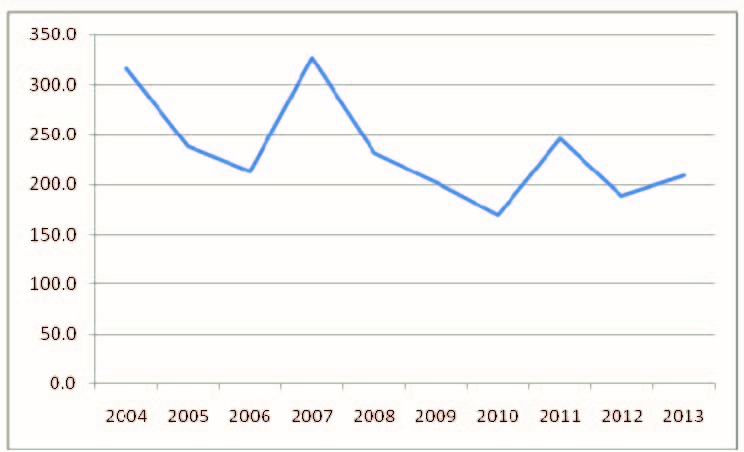
Incidence of varicella in Romania, 2004-2013

The mean incidence of varicella was of 238.2 cases per 100,000 inhabitants, with the highest value of 326.9 cases per 100,000 inhabitants in 2007 and the lowest value of 170.3 cases per 100,000 inhabitants in 2010 (**Fig. 2**).

**Fig. 3:**
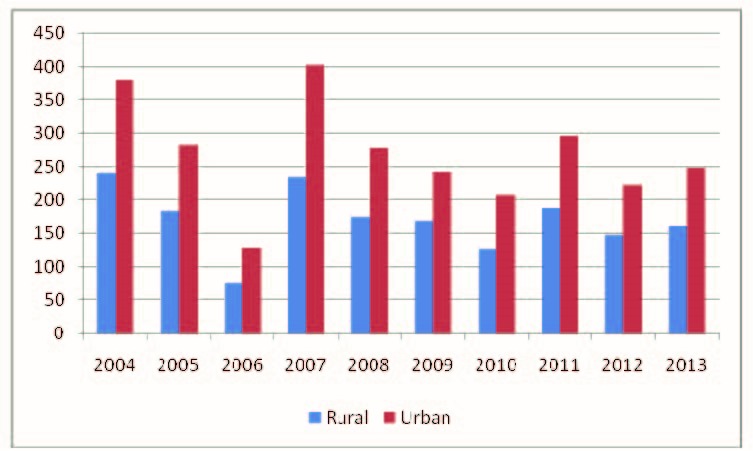
Incidence of varicella cases according to the medium of residence, Romania, 2004-2013

The analysis of cases according to residence showed that cases were reported annually both in urban and in rural areas. The mean incidence in rural areas was of 176.6 cases per 100 000 population, the highest value was recorded in 2004 (240.5 cases per 100 000 population) and the lowest in 2006 (75.9 cases per 100 000 population). The mean incidence in urban areas was 285.04 cases per 100 000 population with the highest value in 2007 (402.2 cases per 100 000 population) and the lowest incidence in 2006 (128.2 cases per 100 000 inhabitants) (**Fig. 3**).

Cases were reported in all age groups throughout the studied period (**Table 1**). The most affected age group was 5-9 years old (mean incidence was 1362.7 cases per 100,000 inhabitants) followed by the age group 1-4 years old (mean incidence was 1297.1 cases per 100,000 inhabitants) and the age group 10-14 years old (mean incidence 947.23 cases per 100,000 inhabitants).

**Table 1:** Incidence of varicella cases per 100 000 inhabitants according to the age group and year, Romania 2004-2013

	**2004**	**2005**	**2006**	**2007**	**2008**	**2009**	**2010**	**2011**	**2012**	**2013**
**< 1 year**	590,3	506,7	507,0	607,4	462,4	811,6	364,0	573,1	454,3	511,4
**1-4 years**	1457,2	1131,1	1130,0	1787,2	1416,5	1223,7	1144,1	1630,9	1277,3	1519,1
**5-9 years**	1735,8	1274,3	1230,2	1910,0	1320,7	1154,4	1050,0	1527,3	1151,7	1301,1
**10-14 years**	1234,9	851,7	865,6	1349,3	881,3	825,5	594,2	909,1	658,0	697,9
**15-19 years**	551,5	399,5	325,3	482,3	324,3	334,0	255,5	385,3	309,7	295,6
**20-24 years**	177,9	141,0	112,4	185,1	126,5	10,9	70,0	92,9	72,9	92,5
**25-34 years**	103,1	86,4	77,7	123,1	90,0	88,2	71,7	96,6	71,9	82,9
**35-44 years**	38,6	35,4	28,6	52,7	39,0	41,0	28,6	47,7	35,8	47,8
**45-54 years**	7,7	5,7	5,5	9,0	6,2	8,0	5,9	8,9	8,4	10,0
**55-64 years**	3,7	2,5	2,9	2,9	3,8	3,0	2,0	3,9	3,4	4,1
**65-74 years**	2,4	1,2	0,9	2,2	1,9	2,5	1,6	1,9	1,9	3,0
**75-84 years**	1,4	0,7	0,1	1,2	1,1	1,1	0,7	1,3	1,9	1,6
**> 84 years**	0,7	0,7	-	0,6	2,4	0,4	0,4	0,8	4,5	5,4

**Fig. 4:**
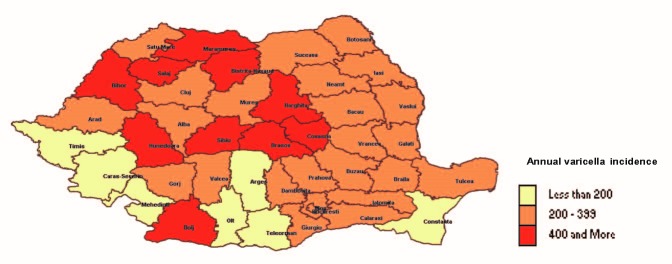
Geographical distribution of cases in Romania, in 2007

**Fig. 5:**
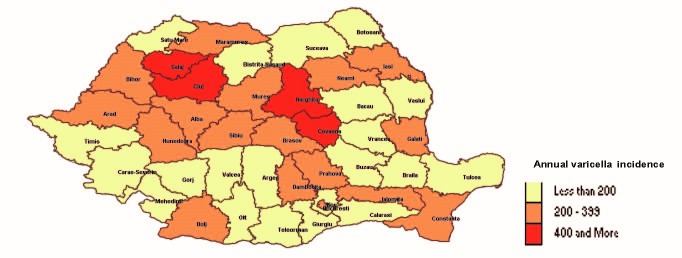
Geographical distribution of varicella cases in Romania, in 2013

The distribution of cases according to the county and year showed that all counties reported a number of cases of varicella (**Fig. 4**,**5**). 

Two deaths from complications were reported in one case in 2008 (25-year-old woman with varicella and pneumonia complications – she was part of a family outbreak with 8 cases, the source of infection being a child of 3 years old) and in 2010 a case (a 32-year-old man with severe bronchopneumonia).

The second study conducted in a hospital, showed that during three years (2011-2013), there were 353 patients hospitalized with varicella; of them, 184 were male (52.1% ) and 169 were female (47.9 %). There were a total of 237/ 353 patients hospitalized (67.1%) from urban areas.

The majority of hospitalized cases 312/ 353 (88.8%) were under 10 years old, as it follows: 157 (44.9%), 1-4 years old, 82 (23.2%), 5-9 years old and 73/ 353 (20.7%), under 1 year old.

Many cases 256/ 353 (72.6 %) attended a community: kindergarten 122/ 353 (34.6%), nursery 83/ 353 (23.5%), school 51/ 353 (14.5%).

The majority of cases 348/ 353 (98.6%) had rash and fever 282/ 353 (79.9%). The other signs and symptoms presented by the patients were cough in 129/ 353 (36.5%), vomiting in 48/ 353 (13.6%), headache in 16/ 353 (4.5%), seizures and drowsiness in 13/ 353 (3.7%), gait disturbance in 10/ 353 (2.8%), dyspnea in 7/ 353 (1.8%), vertigo and edema in 6/ 353 (1.7%).

Only forty-four patients (12.5%) had a medical history (spinal muscular atrophy, stem cell transplant, seizures, epilepsy, asthma, Friedrich's ataxia, beta-thalassemia, TB infection, and CMV infection) and 7 (2%) immunosuppressive diseases: one patient with HIV (0.3%), 5 patients with active cancer (1.4%), one patient with lymphoma (0.3%).

One or more complications occurred in 262 patients (74.2%). The most frequent complications were pneumonia 163/ 262 (46.2%) followed by bacterial skin infection in 57/ 262 (16.1%), hepatitis in 15/ 262 (4.2%), encephalitis 9/ 262 (2.5%), cerebellar ataxia 6 (1.7%), arthritis 4 (1.1%), hemorrhagic varicella 3 (0.8%), thrombocytopenia 3 (0.8%), uveitis 2 (0.7%).

The epidemiological investigation showed that 97 (27.5%) patients had contact with a family member with varicella, 21 patients (5.9%) had contact with a varicella case in a community (kindergartens, schools, maternity) and 231 patients (65.5%) did not know whether they had an infectious contact.

The duration of hospitalization varied from 1 day to 21 days as it follows: 220 (62.3%) patients were admitted for 1-5 days, 106 patients (30%) for 6-10 days, 21 (6%) for 11-15 days and 7 patients (2%) were hospitalized for more than 15 days.

## Discussions

Varicella is one of the most common diseases spread in Romania, with over 36,000 cases reported annually.

In Romania, there is a quarterly mandatory notification system of clinical confirmed cases and deaths, according to age groups and place of residence (aggregated data). The system covers the total country population.

The surveillance systems for varicella and herpes zoster in the European Union are highly heterogeneous or absent because varicella is not included in the list of EU/ EEA priority diseases for surveillance and there is no standard case definition [**8**].

During the 2004 and 2013 period, there were 504,844 cases of varicella investigated in our study, reported at the national level. Basically, around 2.5% of the Romanian population was affected in 10 years. Most cases (70 410 cases) were reported in 2007, when Romania had the highest incidence rate in Europe [**3**].

The mean incidence of varicella was of 238.23 cases per 100 000 population, with the highest value of 326.9 cases per 100,000 inhabitants in 2007, which, according to the statistics, is specific for the countries in Eastern Europe [**8**]. In Western European countries (France, Netherlands, Denmark, UK), the incidence ranged between 300 and 1291/ 100 000 population [**9**-**12**], while in southern Europe (Italy, Spain, Slovenia, Portugal), the incidence ranged between 164 and 1240 cases/ 100,000 inhabitants [**13**-**16**].

The results showed that varicella occurred in all counties and in all age groups, with a higher incidence in children. Most cases (70%) occurred in urban areas and this situation could be explained according to the greater number of specific communities of children and bustle of cities, which are risk factors for airborne transmitted diseases.

During this period, 2 deaths in adults were reported due to complications (pneumonia and severe bronchopneumonia).

Taking into account that mandatory reporting is without information about the clinical, laboratory, evolution, severity and economic impact of varicella, it was necessary to perform a second study in a hospital. The majority of hospitalized patients had the characteristic rash (98.6%) and fever (79.9%), and over 90% of patients were diagnosed with varicella since their admission based on clinical criteria, which showed their diagnostic value. Only a few cases had laboratory confirmation, but laboratory confirmation for varicella was not routinely done. It could be useful in complicated cases and in epidemiological studies [**17**]. After the introduction of vaccination in the National Immunization Program, the suspected cases had to be confirmed by the laboratory in order to monitor the impact of the varicella vaccine program. PCR testing of skin lesions is highly sensitive and specific for the detection of the varicella virus and could be an option [**18**].

Most patients who were hospitalized were in the age group under 10 years (88.8% of cases), where the highest incidence was recorded at the national level.

Of the total cases, 262 patients (74.2%) were hospitalized for having a severe form of illness or had complications during hospitalization, the most common being pneumonia (46.2%). The majority of hospitalizations (85.5%) were found among healthy children, results similar with the studies in Spain, Germany, France, Italy [**19**-**23**].

Most patients (92%) were hospitalized for less than 10 days, as it occurred in the country in Europe where the average length of stay was between 3 and 9 days, the period of hospitalization being dependent on age (longer in adults than in children) and on the presence and type of complications [**8**].

In conclusion, our study results allow us to conclude that varicella is a very common disease in Romania, which may develop complications that can lead to deaths. Although it is a vaccine-preventable disease, vaccination against varicella is not included in the National Immunization Program.

It is very important to introduce a specific surveillance system based on case analysis to provide accurate epidemiological, clinical and laboratory information which will be used to assess whether varicella is a public health problem in Romania and whether the introduction of vaccination in the national immunization program is beneficial. However, given the large number of cases currently in Romania, a solution to this problem could be a sentinel surveillance system type.

**Acknowledgements**

We are grateful to Alina Zaharia and Ruxandra Ștefan for their support in data analysis.
